# Effects of Glyphosate on the Planktonic Microbiota: An Experimental Approach

**DOI:** 10.1002/tox.70019

**Published:** 2026-01-22

**Authors:** Melissa Progênio, Matheus Henrique Oliveira de Matos, Edilaine Corrêa Leite, Bianca Ramos de Meira, João Vitor Bredariol, José Eduardo Gonçalves, Pablo Augusto Poleto Antiqueira, Luiz Felipe Machado Velho

**Affiliations:** ^1^ Núcleo de Pesquisas em Limnologia, Ictiologia e Aquicultura—NUPELIA, Programa de Pós‐graduação em Ecologia de Ambientes Aquáticos Continentais—PEA Universidade Estadual de Maringá—UEM Maringá Brazil; ^2^ Instituto Cesumar de Ciência, Tecnologia e Inovação (ICETI)—UniCesumar Maringá Brazil; ^3^ School of Life Sciences University of Essex Colchester UK

**Keywords:** anthropogenic action, beta diversity, community structure, freshwater, microcosm, pesticides, plankton

## Abstract

Glyphosate is one of the most widely used herbicides in the world, including in Brazil, and its dispersion through habitats and surface waters can impact entire aquatic ecosystems. However, experimental studies evaluating the effects of pesticides on whole planktonic communities, considering attributes such as richness, density and composition—remain scarce. This study evaluated the effects of different glyphosate concentrations on freshwater planktonic microbiota, encompassing cyanobacteria, algae, testate amoebae, autotrophic and heterotrophic nanoflagellates, ciliates, rotifers and copepods. We experimentally simulated four contamination scenarios in freshwater microcosms: (i) control (no glyphosate), (ii) low glyphosate (30 μg/L^−1^), (iii) high glyphosate (500 μg/L^−1^), (iv) the maximum allowed in Brazil (65 μg/L^−1^). The effects of glyphosate varied among biological groups, underscoring the complexity of community‐level responses to contamination. Some groups, such as autotrophic and heterotrophic nanoflagellates, testate amoebae, rotifers, and copepods, responded only in density, with the total microfaunal community following a similar trend. Shifts in species composition were observed for testate amoebae (species replacement) and cyanobacteria (changes in richness), both of which contributed most to beta diversity variation under pesticide exposure. Microcosms exposed to higher glyphosate concentrations exhibited more distinct community compositions compared to the control, suggesting a shift toward more resistant species. These findings highlight the importance of protecting aquatic environments from pesticide contamination, as both individual species and entire communities exhibit differential sensitivity to chemical stressors, potentially affecting ecosystem functioning and services.

## 1 Introduction

The growth of the human population, urbanization and economic development has generated an increasing demand for food [1], which in turn has driven the intensified use of pesticides. A large part of the agricultural products produced by developing countries has high levels of pesticide contamination, causing a serious health risk to the millions of individuals who consume them [2]. The continuous and often uncontrolled application of these synthetic pesticides on agricultural crops has caused an accumulation of toxic residues in the environment over the years, leading to chronic diseases in non‐target organisms [2, 3].

Freshwater ecosystems provide a wide range of ecosystem services to society (i.e., food supply, recreation, climate regulation and participation in biogeochemical cycles) [4]. During atmospheric precipitation or irrigation, and through surface runoff or even indirectly through percolation in the soil, pesticides from agricultural activities can directly impact water bodies, reaching groundwater as well [5]. These environments are suffering constant anthropogenic pressures and are highly vulnerable to environmental changes, such as the presence of pesticides, with an increasing risk of biodiversity loss [6, 7]. Aquatic communities are directly and indirectly affected by the presence of pesticides in the water. For example, some substances can reduce the abundance of periphytic algae and zooplankton [8]. In addition, pesticide contamination can alter primary production rates and disrupt food web components [9].

Glyphosate is one of the most widely used herbicides in the world [10], including Brazil. First introduced in 1974 as a post‐emergence, non‐selective herbicide, glyphosate has gained popularity due to its high efficacy, becoming a dominant herbicide globally [11]. Its widespread use in agriculture, forestry, urban areas, and aquaculture facilitates its dispersion through ecosystems, including surface waters, affecting plants, animals, and food webs [12]. Glyphosate exposure has been shown to reduce species richness and diversity of aquatic organisms, especially planktonic communities, leading to shifts in community structure [13].

Several studies have demonstrated the potential of glyphosate to alter biological processes in planktonic organisms. In microalgae, for example, significant changes in growth, cell volume, and pigment content were observed in *Scenedesmus vacuolatus* [14]. Similarly, zooplankton species such as *Daphnia spinulata* showed negative responses to glyphosate exposure, with a reduction in the number of offspring and broods over time [15]. Additionally, glyphosate can interfere with the hatching of zooplankton species from egg banks, promoting changes in community structure, especially when associated with other pesticides [16, 17, 18]. It is important to note that these studies were conducted at the species level, in which specific responses are directly associated with the presence of glyphosate. As highlighted by Lozano [19], analyses based solely on abundance and species richness may be insufficient to fully capture the real impact of herbicides, as important changes in species interactions and community composition can be masked. Thus, to more comprehensively assess the effects of pesticides such as glyphosate on community structure and dynamics, measures of alpha diversity (local diversity) and beta diversity (variation in species composition among environments) can be employed. Depending on the intensity of environmental stress, the components of beta diversity—specifically, species replacement and richness difference [20]—play complementary roles in revealing the mechanisms that structure ecological communities [21].

The aim of this study was to test the effects of different concentrations of the pesticide glyphosate on the structure of planktonic microbiota communities using an experimental approach. Thus, the present study aims to evaluate the effect of glyphosate at the community level, considering not only the direct action of the stressor on the environment but also the simultaneous interactions between different species that alter the dynamics of the environment, presenting a more realistic response to the environmental impacts. Specifically, we formulated the following hypotheses regarding glyphosate addition: (i) we expected a reduction in species richness (alpha diversity) and density of groups over time, particularly in treatments with higher glyphosate concentrations; (ii) the species composition will change over time in treatments with the presence of glyphosate, especially in higher concentrations, which would be reflected by an increase in beta diversity due to species loss (Richness difference, or βrich).

## 2 Materials and Methods

### 2.1 Experimental Design

The water samples used for the experiment were collected from the Baía River (22°43′ S; 53°17′ W), located on the right margin of the Paraná River in the state of Mato Grosso do Sul, Brazil. Samples were collected in April 2023, and 100 L of water was collected from the subsurface (approximately 40 cm below) of the river and transported to the laboratory for acclimatization for 3 days.

A total of 60 artificial microcosms (700 mL) were used, in sterile 1 L bottles. The microcosms were randomly distributed in five blocks per sampling time, with the treatments randomized within each block. To verify the effect of the different treatments over time of the planktonic microbiota (cyanobacteria, algae, auto/heterotrophic nanoflagellates, ciliates, testate amoebae, copepods and rotifers), the experiment had a duration a total of 7 days, subdivided into three sampling periods: T1 (day one, start of the experiment), T3 (day 3 of the experiment) and T7 (day 7, end of the experiment). All treatments, at each time, were collected after the addition of the pesticide. The experiment duration (7 days) was selected to capture early community responses to glyphosate exposure (changes in richness, density, and species composition) while avoiding longer‐term confounding processes such as long‐term adaptation or secondary successional replacement. Short‐term mesocosm assays are a standard approach to detect pulse effects and immediate community responses in planktonic systems [22].

**FIGURE 1:**
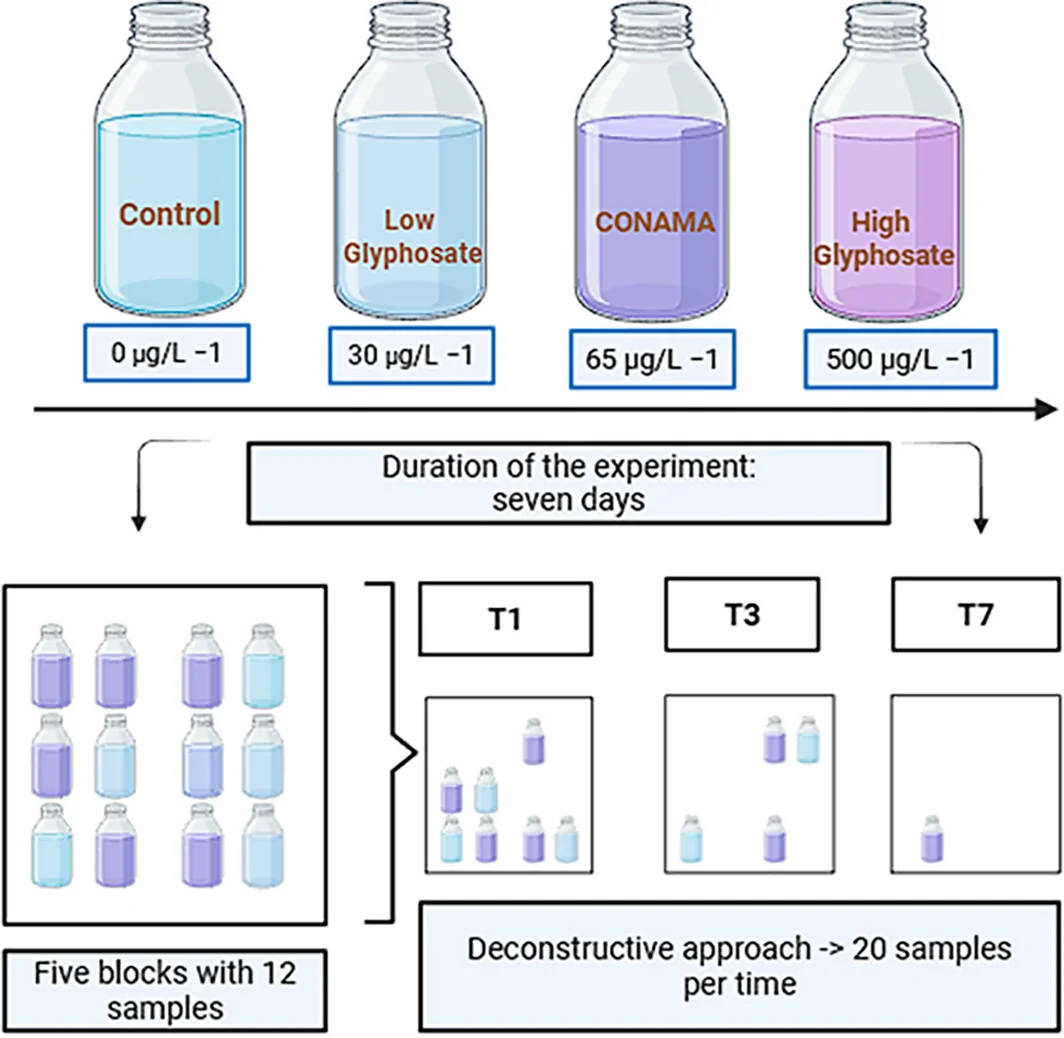
Diagram of the experimental design: 60 experimental units subdivided into 5 randomized blocks, with 4 treatments each and 5 replicates of each treatment, during 3 sampling times (T1, T3, T7). The lower part of the figure indicates the deconstructive approach used in this experiment. The concentrations indicate respectively: Control; water without glyphosate, Low glyphosate 30 μg L^−1^, CONAMA concentration 65 μg L^−1^, and High glyphosate 500 μg L^−1^.

The microcosms were incubated on an agitator under a 12:12 photoperiod at 25°C with continuous agitation. To evaluate the effects of different glyphosate concentrations on planktonic microbiota structure, an experimental approach was used to simulate four glyphosate contamination scenarios (Figure 1). The following treatments were applied in five replicates to the microcosms: (i) control (water without glyphosate), (ii) low glyphosate (30 μg/L^−1^), (iii) high glyphosate (500 μg/L^−1^), (iv) CONAMA glyphosate (maximum concentration allowed by CONAMA in the aquatic environment—65 μg/L^−1^) [23] (National Environment Council) is a Brazilian regulatory agency.

### 2.2 Sampling and Laboratorial Analysis

The experiment was sampled in a deconstructive method, where 20 microcosms were completely removed from the experiment at each experimental time to collect the planktonic microbiota. The following communities were sampled: testate amoebae, ciliates protists, nanoflagellates, rotifers, copepods, cyanobacteria, and algae. To obtain the planktonic microbiota, the total volume (700 mL) of the bottles was filtered using a 10‐μm net and concentrated into 100 mL. The samples were then fixed with alkaline lugol, formalin, and sodium thiosulphate [24] and preserved in the dark until the organisms were counted. For the algae and cyanobacteria, 100 mL samples were obtained in their totality and fixed in alkaline lugol. Because samples were fixed and preserved for later counting, the viability of individuals could not be assessed directly. Therefore, abundance and biomass reported here refer to the total number of individuals preserved in the samples. Therefore, changes in abundance are interpreted as population declines that may result from mortality, reduced reproduction, or other processes.

The algae and cyanobacteria density and richness were estimated using an inverted microscope, using the Utermöhl method [25], with a 40× magnification. Each 15 mL sample was diluted to 5 mL or more, according to the concentration of detritus. The sedimentation volume was 5 mL, and the sedimentation time was at least 3 h. A count of 50 fields was performed randomly. Density values were calculated according to APHA [26], and species were identified according to specific literature [27].

To analyze part of the planktonic microfauna (testate amoebae, ciliates, protists, rotifers, and copepods), the samples were colored with Rose Bengal for direct analysis using an optical microscope with a 20× objective. Microfauna density was estimated by counting the individuals in Sedgewick‐Rafter chambers. All samples were quantified in full. The organisms were identified to the smallest possible taxonomic level using specific literature [28, 29, 30, 31, 32].

To determine the density and biomass of autotrophic and heterotrophic nanoflagellates present in the different treatments, 15 mL of water samples were collected. This volume of water was placed in Falcon tubes, and the samples were fixed with glutaraldehyde at 2% final concentration [33]. After fixation, the samples were stored at 4°C in a refrigerator. To estimate the density (cells mL^−1^) and biomass of the nanoflagellates, the water samples were filtered through a Nucleopore/Watchman black polycarbonate filter with a pore opening of 0.8 μm and a diameter of 25 mm, and colored with 0.1% 4′,6‐diamidino‐2‐phenylindol (DAPI) for 15 min in the dark [34]. After filtering, slides containing the filters were prepared and saved in a −18°C freezer. The density of the nanoflagellates was determined simultaneously using UV light, and the autotrophic nanoflagellates were separated from the heterotrophic by the red color reflected when these organisms are exposed to green light, due to the presence of chlorophyll. Fifty fields were counted per sample using an epifluorescence microscope (Olympus model BX51) at 1000× magnification.

### 2.3 Data Analysis

To evaluate how the biomass of nanoflagellates (both heterotrophic and autotrophic), and the species richness and total density of the planktonic microbiota were affected by the predictor variable (glyphosate) (hypothesis i), for each time (T1, T3 and T7), Linear Mixed Effects Models (LME) were used, function “lmer” from the lme4 package [35], with glyphosate and time as fixed effects, and blocks as a random effect. Additionally, an Analysis of Variance (ANOVA) was conducted using the linear mixed‐effects model using the “anova.lme” function to examine temporal and treatment differences. The assumptions of linearity and homoscedasticity were tested. In cases with significant results, Tukey's post hoc test was applied using the “emmeans” package to assess differences between treatments, considering *p*‐values < 0.05 significant.

To evaluate how glyphosate altered, over time, total beta diversity and its two components of replacement (βrepl) and difference in richness (βrich), the Jaccard distance matrix was analyzed with the method proposed by Podani & Schmera [20], using the “beta” function of the BAT package [36]. To determine any treatment related patterns in total beta diversity and its components, a distance‐based redundancy analysis (dbRDA; [37]) was applied using the “dbrda” function of the vegan package [38]. This analysis allowed us to test the significance of the associations. Also, the biodiversity of the communities was analyzed using the Shannon‐Weaver diversity index [39] of the “vegan” package. The graphics were made using the ggplot2 package. All analyses were done using the R statistical analysis software (version 4.1.2; [40]).

## 3 Results

### 3.1 Density, Richness, Shannon Diversity and Biomass (Nanoflagellates)

**FIGURE 2:**
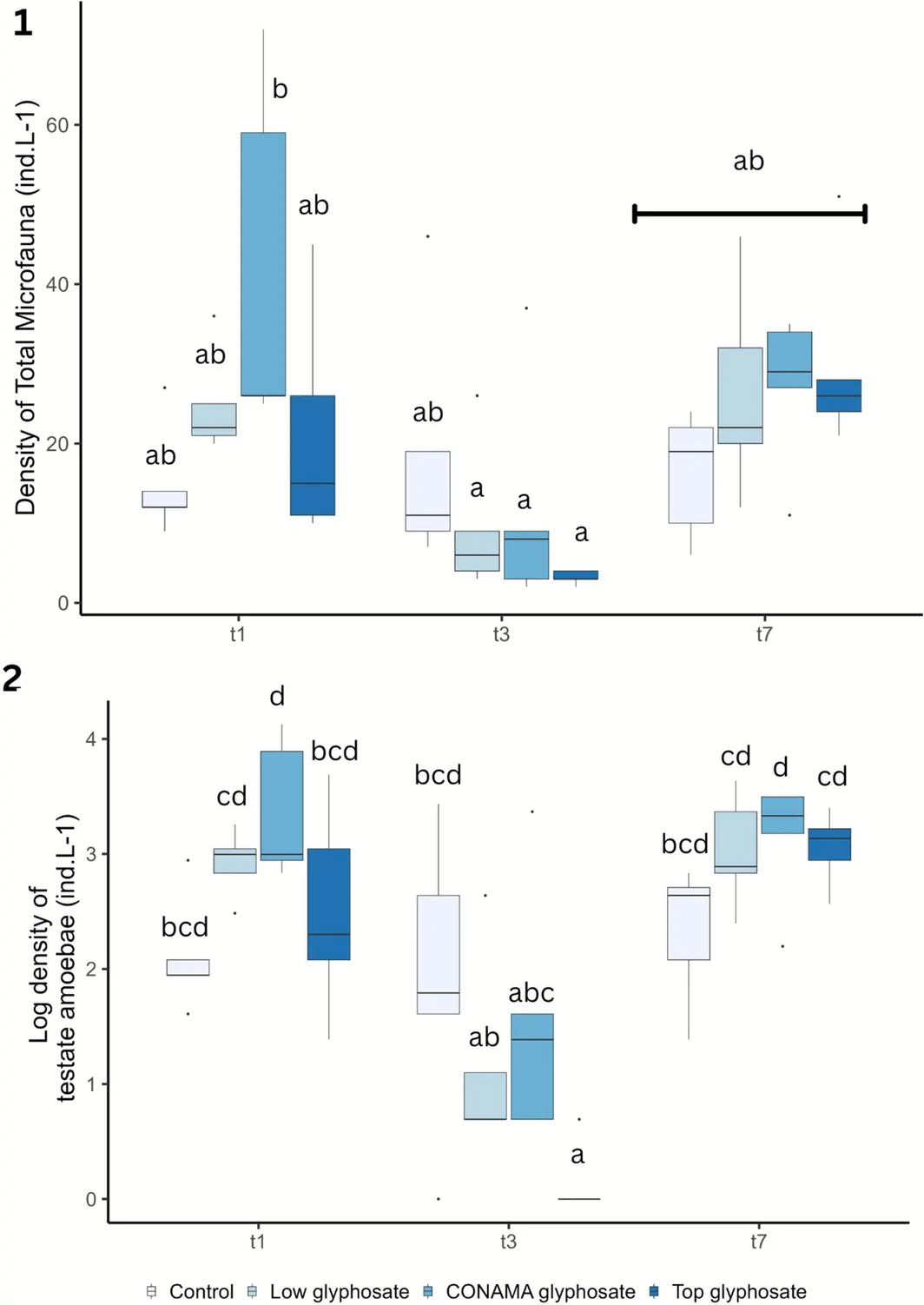
(1) Total density of microfauna at different pesticide concentrations over time. (2) Total richness of testate amoebae at different pesticide concentrations over time. (Control: Without glyphosate; LG: Low glyphosate, 30 L^−1^; CN: CONAMA concentration, 65 L^−1^; HG: High glyphosate, 500 L^−1^). The letters indicate the differences between treatments.

The components of planktonic microbiota exhibited distinct responses to glyphosate exposure over time (Table 1). The total microfauna density showed a significant reduction between T1 and T3, particularly when the CONAMA/T1 treatment was compared with the Low, CONAMA and Top glyphosate treatments at T3 (Figure 2(1)).

**TABLE 1:** The effects of glyphosate over time on the density and richness of planktonic microbiota.

Effect	df	*F*	*p*
*Density*			
Autotrophic nanoflagellates			
Pesticide*Time	6	2.776	**0.022**
Pesticide	3	15.117	**< 0.01**
Time	2	15.724	**< 0.01**
Heterotrophic nanoflagellates			
Pesticide*Time	6	1.393	0.239
Pesticide	3	11.847	**< 0.01**
Time	2	9.306	**< 0.01**
Cyanobacteria			
Pesticide*Time	6	0.368	0.895
Pesticide	3	0.794	0.504
Time	2	5.371	**< 0.01**
Algae			
Pesticide*Time	6	0.843	0.543
Pesticide	3	2.109	0.113
Time	2	3.354	**0.044**
Total microfauna			
Pesticide*Time	6	2.359	**0.046**
Pesticide	3	2.037	0.122
Time	2	9.270	**< 0.01**
Ciliates			
Pesticide*Time	6	0.853	0.537
Pesticide	3	1.088	0.364
Time	2	9.776	**< 0.01**
Testate amoebae			
Pesticide*Time	6	3.248	**< 0.01**
Pesticide	3	3.371	0.027
Time	2	34.357	**< 0.01**
Rotifera			
Pesticide*Time	6	3.464	**< 0.01**
Pesticide	3	0.070	0.975
Time	2	0.003	0.996
Copepoda			
Pesticide*Time	6	3.288	**< 0.01**
Pesticide	3	0.608	0.613
Time	2	1.197	0.312
*Richness*			
Cyanobacteria			
Pesticide*Time	6	0.999	0.438
Pesticide	3	2.035	0.123
Time	2	3.414	**0.042**
Algae			
Pesticide*Time	6	1.023	0.423
Pesticide	3	1.910	0.0142
Time	2	11.406	**< 0.01**
Total microfauna			
Pesticide*Time	6	1.442	0.221
Pesticide	3	2.518	0.07
Time	2	21.431	**< 0.01**
Ciliates			
Pesticide*Time	6	0.603	0.726
Pesticide	3	2.238	0.097
Time	2	9.092	**< 0.01**
Testate amoebae			
Pesticide*Time	6	1.986	0.088
Pesticide	3	2.776	0.052
Time	2	48.160	**< 0.01**
Rotifera			
Pesticide*Time	6	1.810	0.119
Pesticide	3	1.006	0.399
Time	2	4.683	**0.014**
*Biomass*			
Autotrophic nanoflagellates			
Pesticide*Time	6	0.819	0.561
Pesticide	3	5.772	**< 0.01**
Time	2	4.869	**0.012**
Heterotrophic nanoflagellates			
Pesticide*Time	6	0.649	0.691
Pesticide	3	6.327	**< 0.01**
Time	2	0.308	0.736

*Note:* Values in bold represent significant relationships with *p* < 0.05.

**FIGURE 3:**
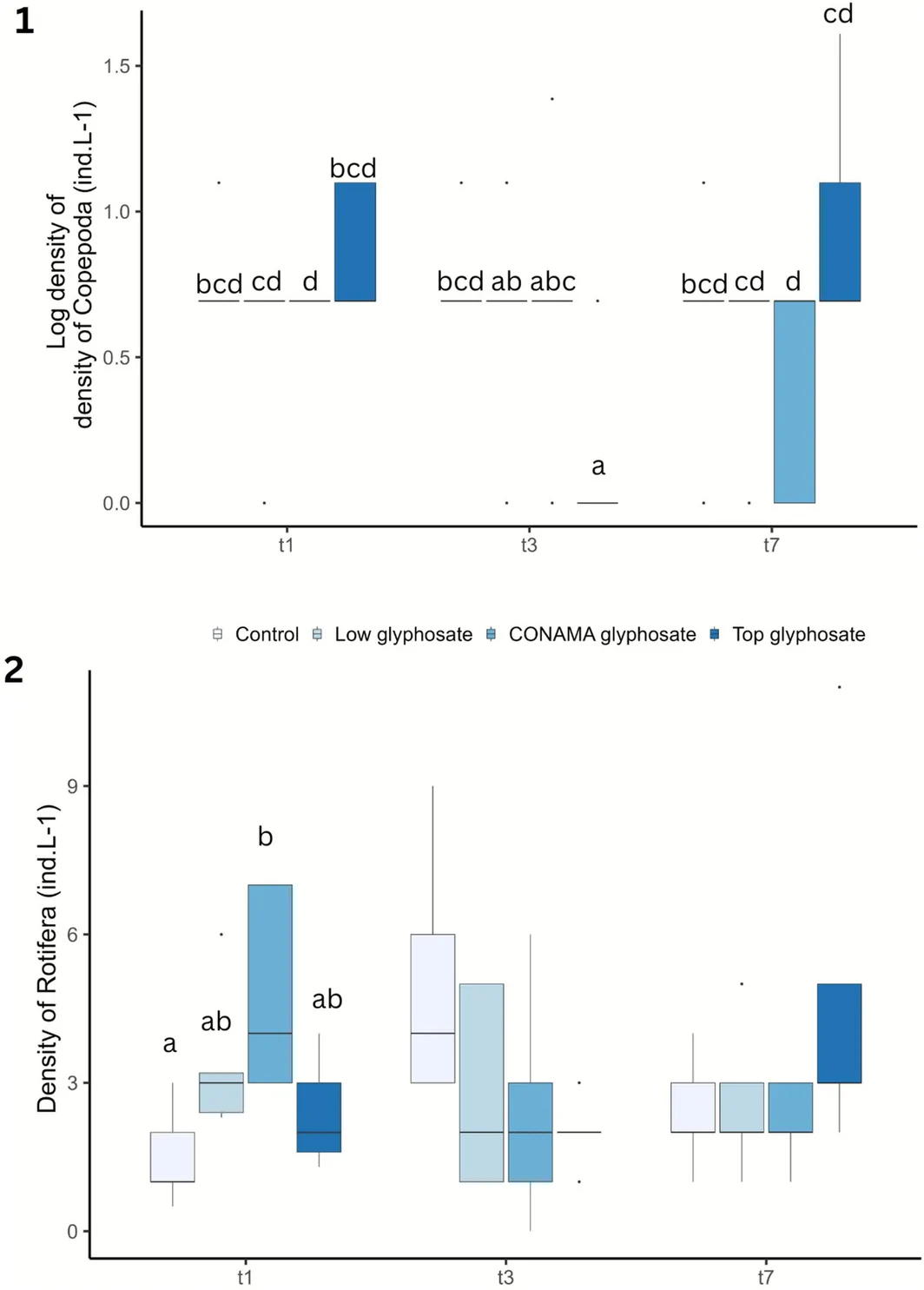
(1) Total density of copepods at different pesticide concentrations over time. (2) Total richness of rotifers at different pesticide concentrations over time. (Control: Without glyphosate; LG: Low glyphosate, 30 μg L^−1^; CN: CONAMA concentration, 65 μg L^−1^; HG: High glyphosate, 500 μg L^−1^).

Testate amoebae density exhibited an oscillatory response, with a reduction at T3 across all glyphosate treatments, followed by recovery at T7 (Figure 2(2)). A similar, though less pronounced, trend was observed for copepods, which showed lower densities in the Top glyphosate treatment at T3 and in the CONAMA treatment at T1 and T7(Figure 3(1)). For rotifers, no clear temporal trend was detected, but the interaction between time and glyphosate was significant. At T1, the control exhibited lower values compared with the CONAMA treatment (Figure 3(2)).

**FIGURE 4:**
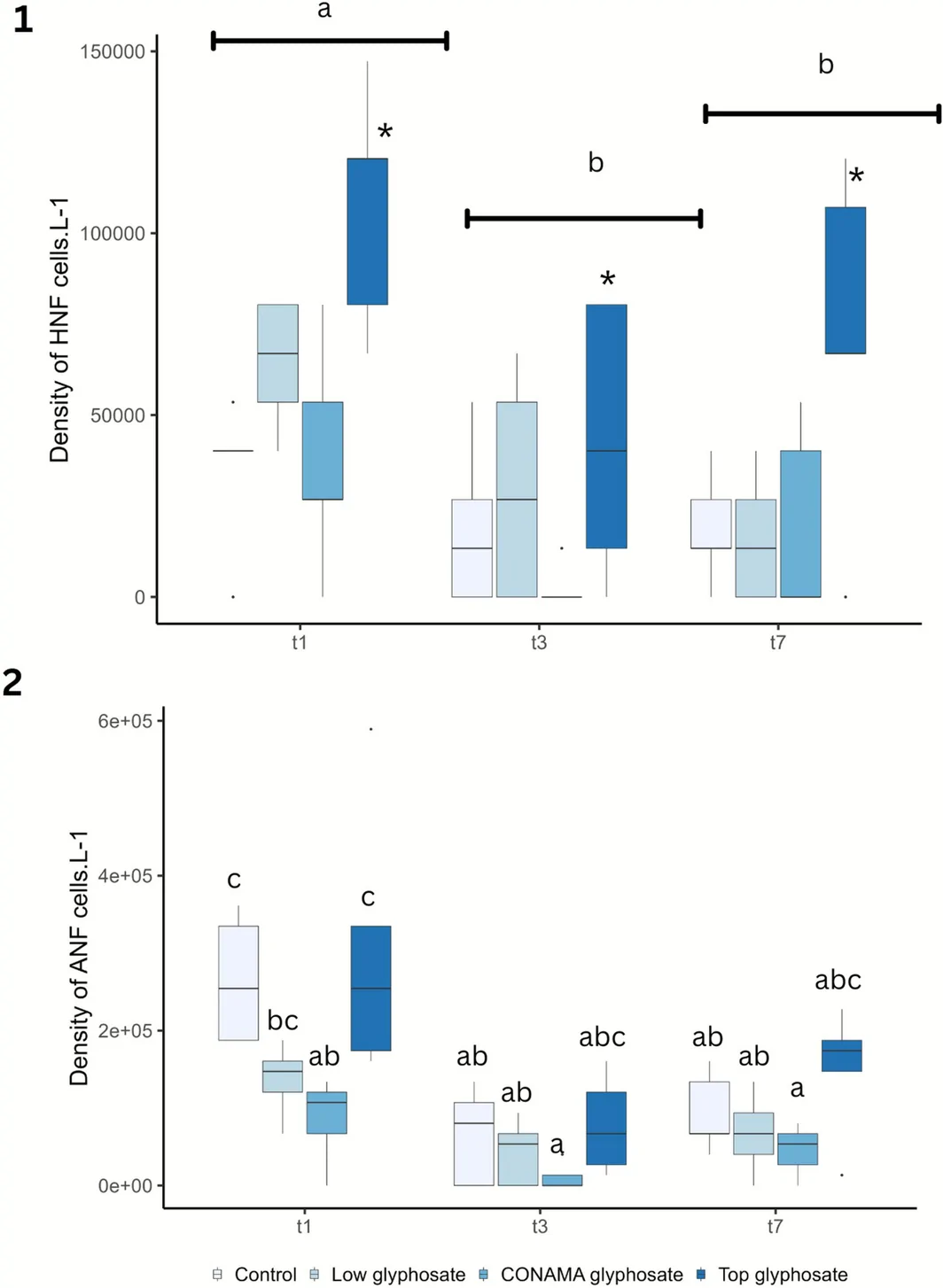
(1) Density of heterotrophic nanoflagellates (HNF) at different pesticide concentrations over time. (2) Density of autotrophic nanoflagellates (ANF) at different pesticide concentrations over time. (Control: Without glyphosate; LG: Low glyphosate, 30 μg L^−1^; CN: CONAMA concentration, 65 μg L^−1^; HG: High glyphosate, 500 μg L^−1^). The letters indicate the differences between treatments.

**FIGURE 5:**
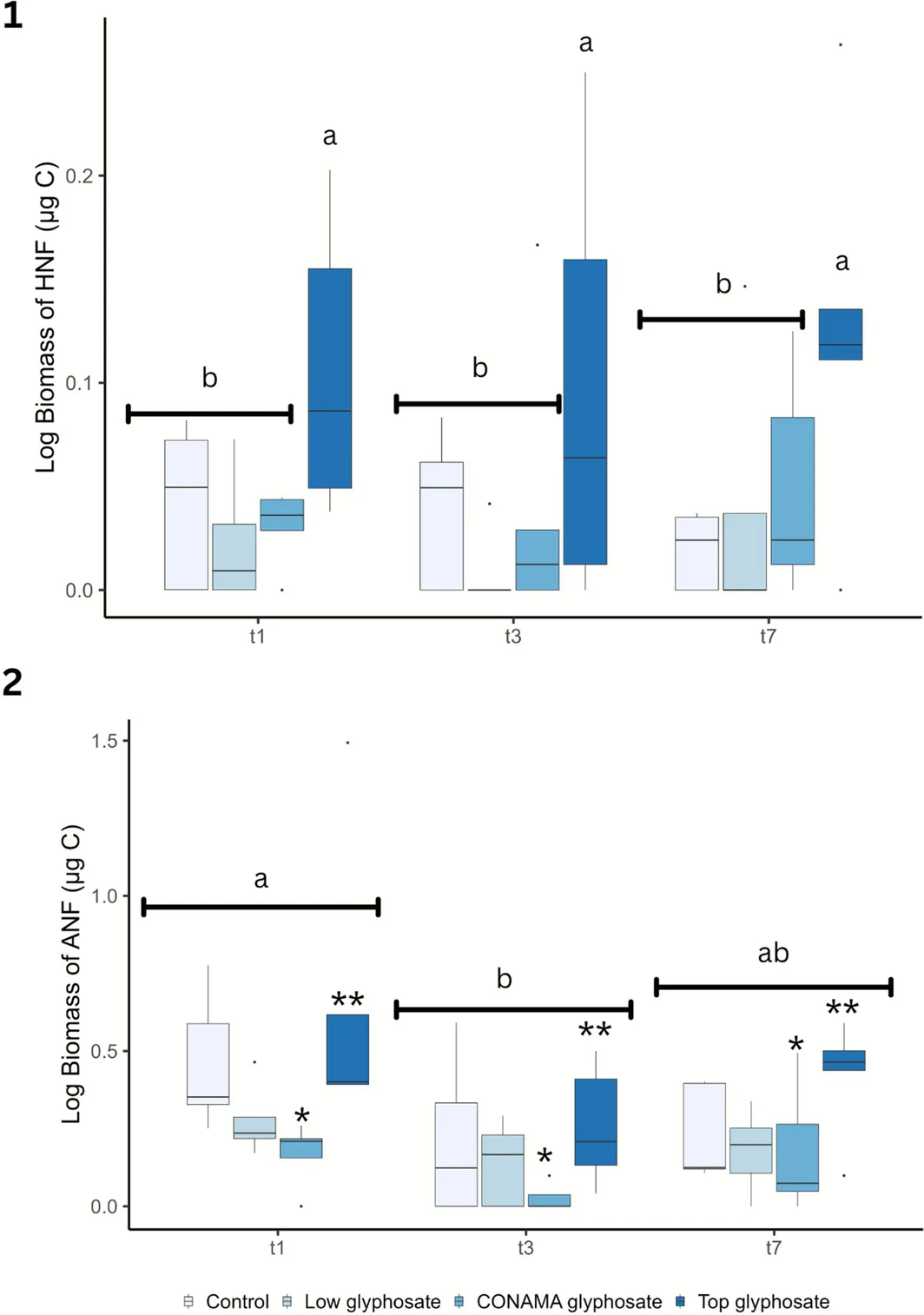
(1) Biomass of heterotrophic nanoflagellates (HNF) at different pesticide concentrations over time. (2) Biomass of autotrophic nanoflagellates (ANF) at different pesticide concentrations over time. (Control: Without glyphosate; LG: Low glyphosate, 30 μg L^−1^; CN: CONAMA concentration, 65 μg L^−1^; HG: High glyphosate, 500 μg L^−1^). When there is not interaction between treatment and time: The letters indicate the differences between time and the * indicate differences between treatments.

Heterotrophic nanoflagellates (HNF) showed a general decrease in density over time, except in the top glyphosate treatment, where densities were higher than in the other treatments (Figure 4(1)). In contrast, autotrophic nanoflagellates (ANF) showed a progressive increase throughout the experiment, with slightly lower values in CONAMA/T1 and T7, but an overall trend of growth in the presence of glyphosate (Figure 4(2)). For biomass, there was an increase in HNF biomass in the top glyphosate treatment (Figure 5(1)). For ANF biomass, the predictor variables responded separately. There was a reduction over time until T3, followed by constancy at T7. Meanwhile, among treatments, there was a difference in CONAMA and top glyphosate, which presented higher values (Figure 5(2)).

**FIGURE 6:**
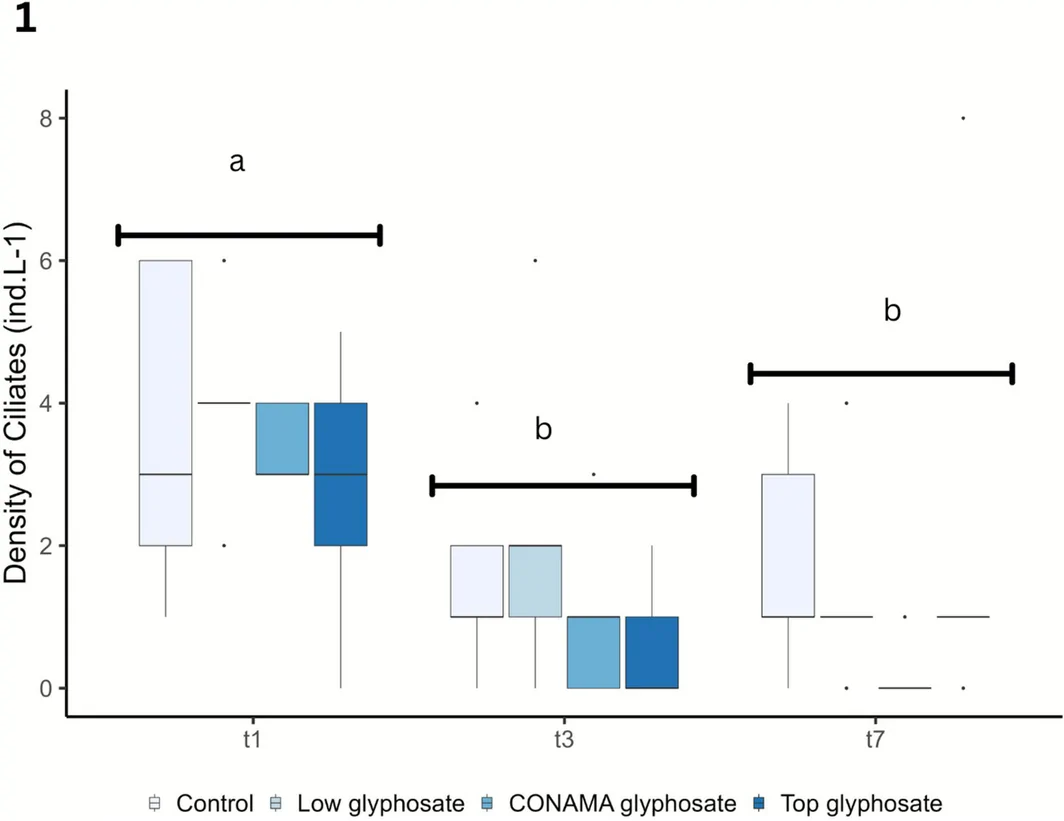
Total density of ciliates at different pesticide concentrations over time (Control: Without glyphosate; LG: Low glyphosate, 30 μg L^−1^; CN: CONAMA concentration, 65 μg L^−1^; HG: High glyphosate, 500 μg L^−1^). The letters indicate the differences between treatments.

**FIGURE 7:**
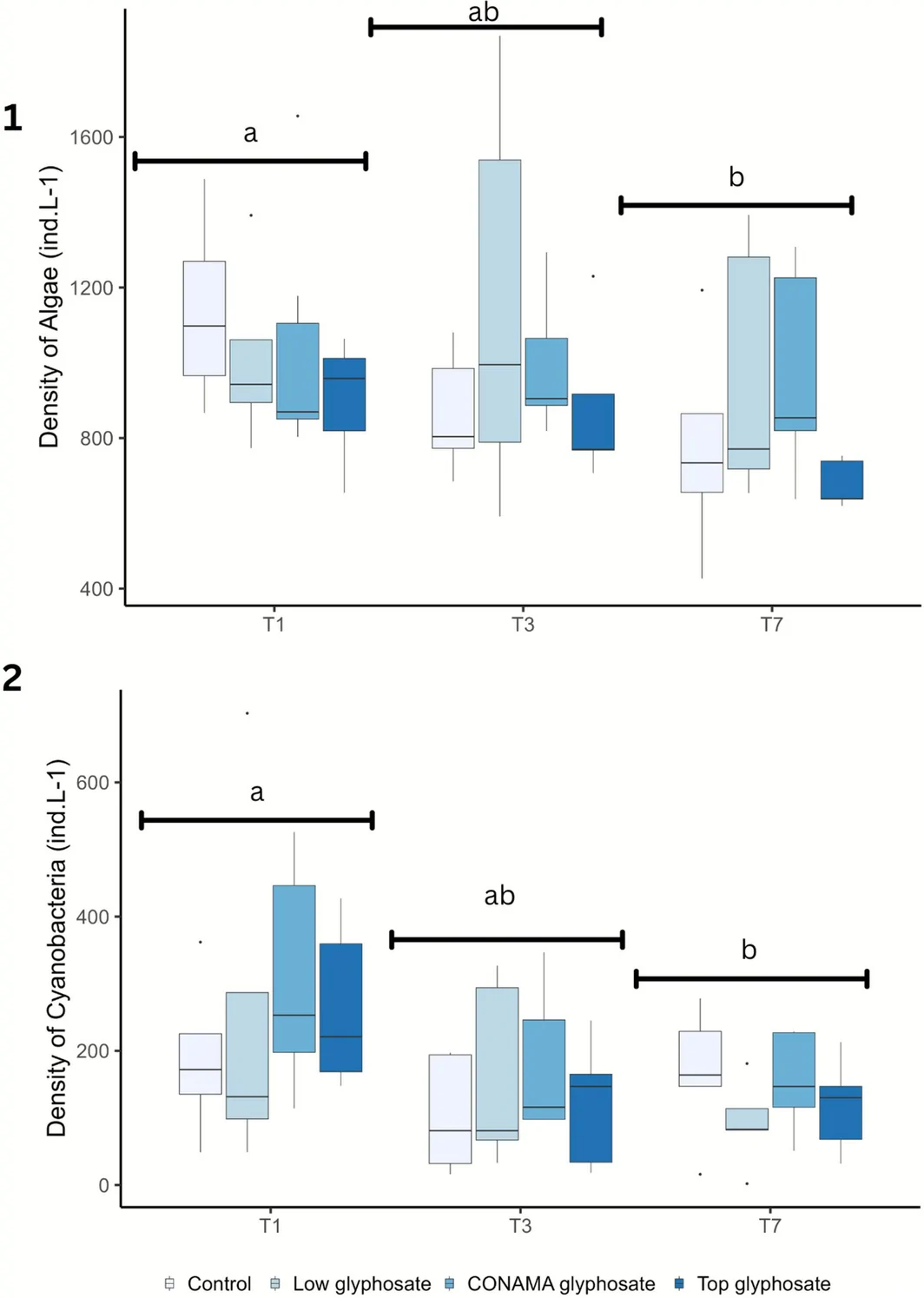
(1) Density of algae at different pesticide concentrations over time. (2) Density of cyanobacteria at different pesticide concentrations over time. (Control: Without glyphosate; LG: Low glyphosate, 30 μg L^−1^; CN: CONAMA concentration, 65 μg L^−1^; HG: High glyphosate, 500 μg L^−1^). The letters indicate the differences between treatments.

**FIGURE 8:**
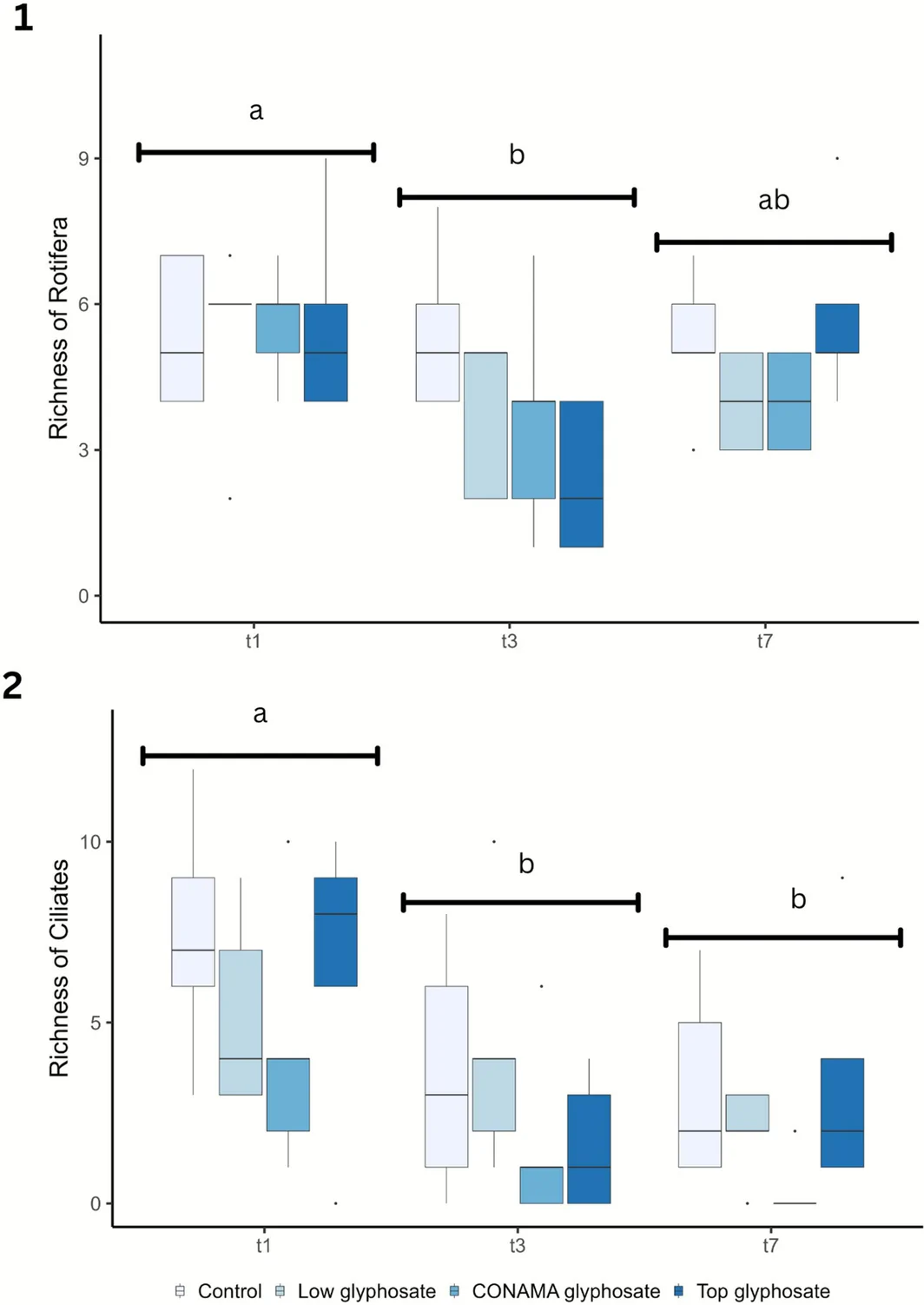
(1) Richness of ciliates at different pesticide concentrations over time. (2) Richness of rotifers at different pesticide concentrations over time. (Control: Without glyphosate; LG: Low glyphosate, 30 L^−1^; CN: CONAMA concentration, 65 L^−1^; HG: High glyphosate, 500 L^−1^). The letters indicate the differences between treatments.

Other groups exhibited density changes only over time. Ciliates, algae, and cyanobacteria presented their highest densities at T1, generally decreasing toward T7 (Figures 6 and 7(1/2), respectively). Similarly, the richness (alpha diversity) varied significantly over time for ciliates, rotifers, algae, and cyanobacteria, showing a continuous decline across sampling periods (Figures 8(1/2) and 9(1/2), respectively). In contrast, total microfauna and testate amoebae richness displayed oscillatory dynamics, with initial decreases followed by increases over time (Figure 10(1/2), respectively).

**FIGURE 9:**
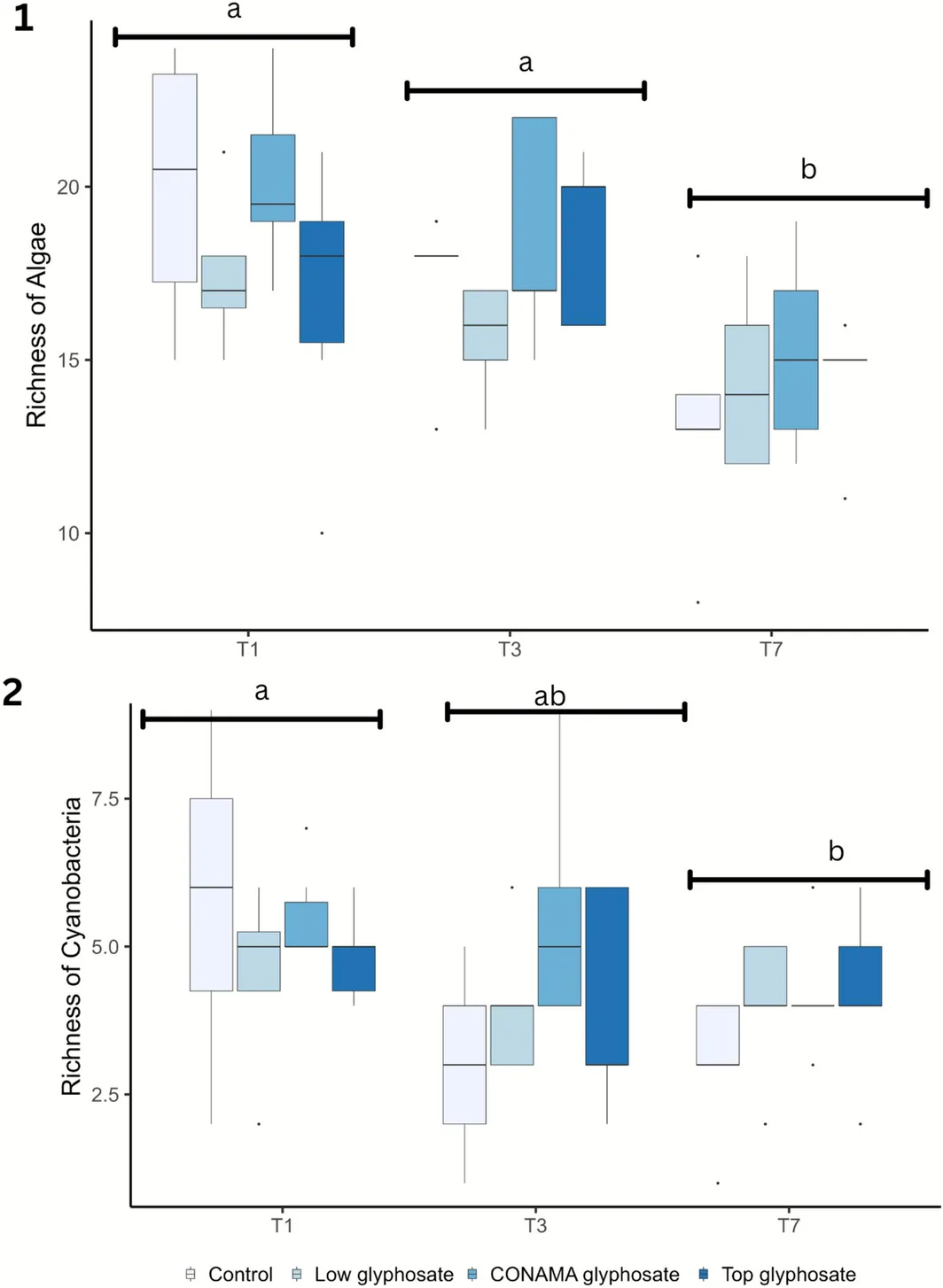
(1) Richness of algae at different pesticide concentrations over time. (2) Richness of cyanobacteria at different pesticide concentrations over time. (Control: Without glyphosate; LG: Low glyphosate, 30 μg L^−1^; CN: CONAMA concentration, 65 μg L^−1^; HG: High glyphosate, 500 μg L^−1^). The letters indicate the differences between treatments.

**FIGURE 10:**
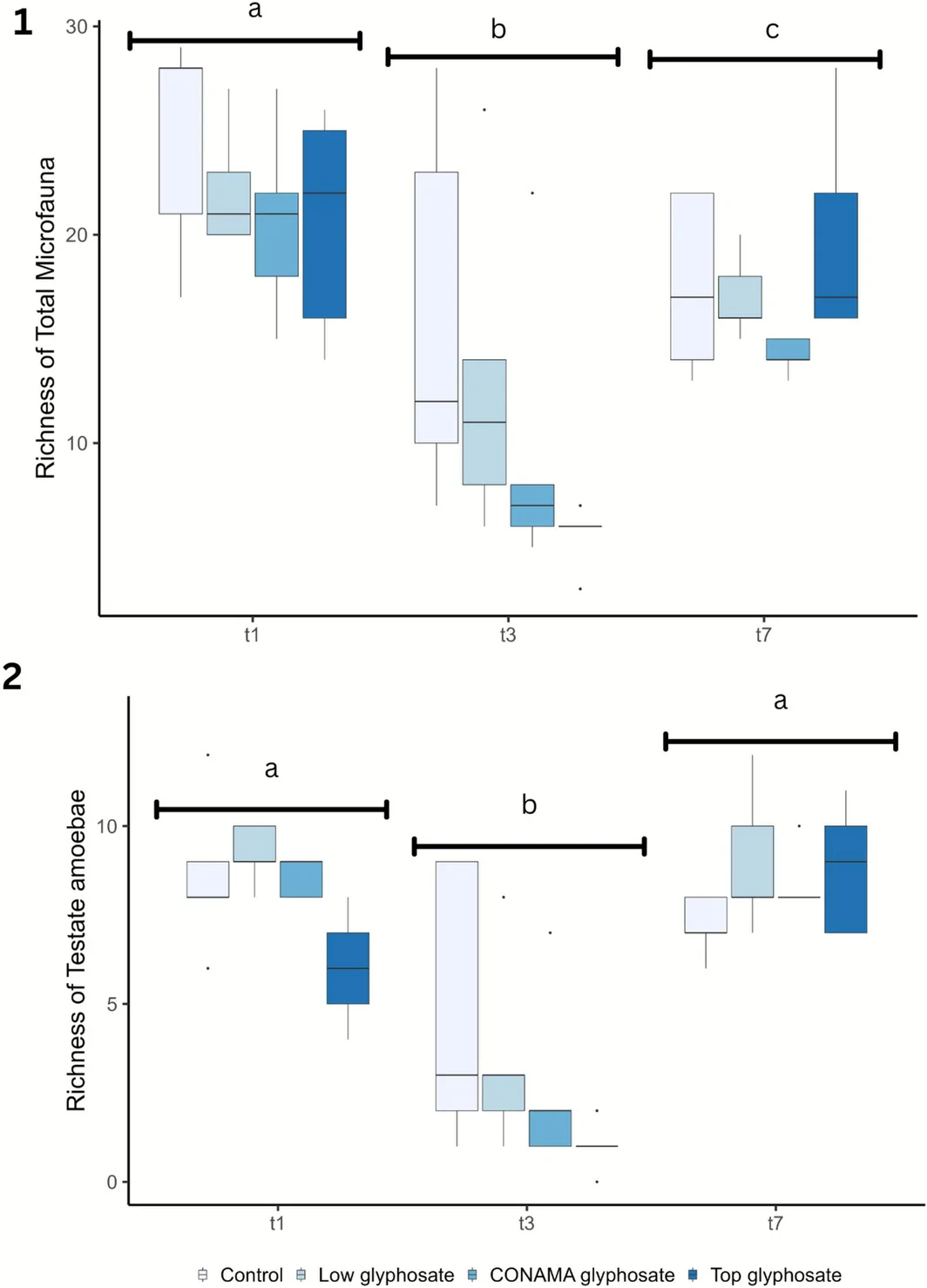
(1) Richness of total microfauna at different pesticide concentrations over time. (2) Richness of testate amoebae at different pesticide concentrations over time. (Control: Without glyphosate; LG: Low glyphosate, 30 μg L^−1^; CN: CONAMA concentration, 65 μg L^−1^; HG: High glyphosate, 500 μg L^−1^). The letters indicate the differences between treatments.

The Shannon diversity index (*H*′) results evidenced variations between the autotrophic and heterotrophic fractions. We observed a reduction in the diversity of heterotrophic organisms (total microfauna, ciliates, testate amoebae, rotifers, and especially for copepods) as glyphosate concentrations increased in the treatments (Table 2), while the autotrophic organisms (cyanobacteria and algae) showed a slight increase in diversity at higher pesticide concentrations (Table 2).

**TABLE 2:** The Shannon index in different concentrations of glyphosate.

Groups	Control	Low glyphosate	CONAMA glyphosate	Top glyphosate
Cyanobacteria	1.111	1.169	1.455	1.405
Algae	2.204	1.979	2.204	2.234
Total Microfauna	2.433	2.326	1.979	2.142
Ciliates	1.127	1.019	0.501	0.969
Testate Amoebae	1.407	1.434	1.197	1.054
Rotifers	1.483	1.212	1.248	1.237
Copepods	0.808	0.597	0.463	0.436

### 3.2 Beta Diversity

**FIGURE 11:**
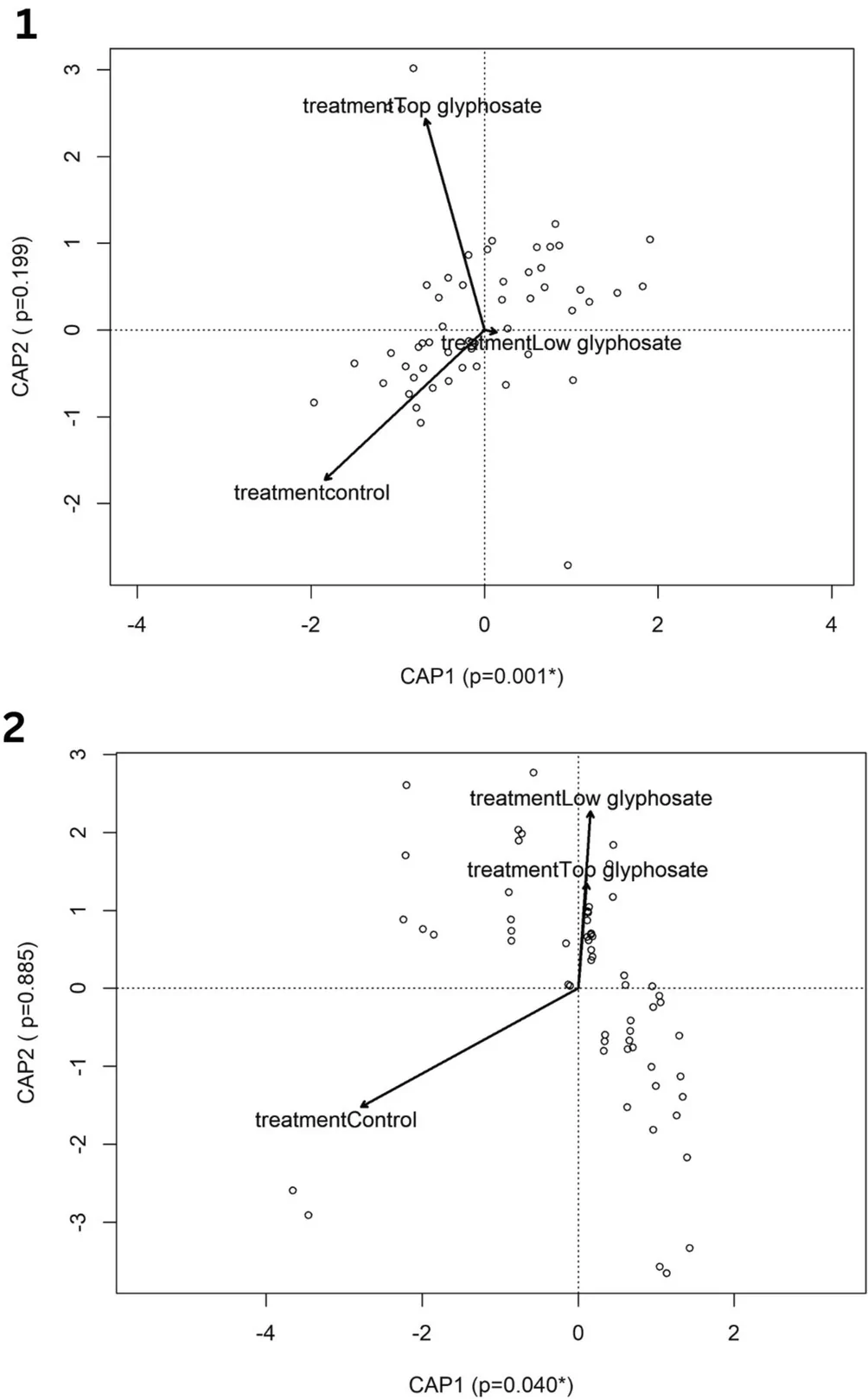
(1) Distance‐based redundancy analysis for *β*_repl_ of the testate amoebae. (2) *β*_rich_ of the cyanobacteria the dissimilarity response between treatments in the glyphosate. The dbRDA1 and dbRDA2 represent the first and second constraint axes generated by the analysis, respectively.

In relation to total beta diversity, glyphosate altered the testate amoebae composition, and these changes were determined specially by species substitution (βrepl) (Figure 11(1)). Differently, the significant changes observed to cyanobacteria were due to the difference of richness (βrich) (Table 3) (Figure 11(2)), the control treatment being different from the treatments with the addition of glyphosate.

**TABLE 3:** The effects of glyphosate over time on dissimilarity of planktonic microbiota composition.

Effect	df		*F*	*p*
*Cyanobacteria*				
βtotal				
Time	2		2.734	**0.001**
Pesticide	3		0.973	0.506
βrepl				
Time	2		2.191	**0.003**
Pesticide	3		0.919	0.613
βrich				
Time	2		1.187	0.059
Pesticide	3		2.086	**0.035**
*Algae*				
βtotal				
Time	2		3.161	**0.001**
Pesticide	3		1.088	0.257
βrepl				
Time	2		2.220	**0.001**
Pesticide	3		1.025	0.534
βrich				
Time	2		7.174	**0.001**
Pesticide	3		1.183	0.286
*Ciliates*				
βtotal				
Time	2		1.599	0.103
Pesticide	3		0.888	0.679
βrepl				
Time	2		1.107	0.337
Pesticide	3		0.981	0.508
βrich				
Time	2		5.755	**0.004**
Pesticide	3		1.188	0.298
*Testate Amoebae*				
βtotal				
Time	2		3.861	**0.002**
Pesticide	3		1.333	0.149
βrepl				
Time	2		4.000	**0.001**
Pesticide	3		1.646	**0.010**
βrich				
Time	2		1.312	0.245
Pesticide	3		0.566	0.763
*Rotifera*				
βtotal				
Time	2		2.840	**0.001**
Pesticide	3		1.172	0.202
βrepl				
Time	2		2.459	**0.002**
Pesticide	3		1.265	0.097
βrich				
Time	2		0.706	0.555
Pesticide	3		1.055	0.349

*Note:* Values in bold represent significant relationships with *p* < 0.05.

## 4 Discussion

### 4.1 Density, Richness, Shannon Diversity and Biomass (Nanoflagellates)

The effect of the pesticide varied according to the biological group analyzed, highlighting the complexity of the planktonic microbiota's responses to glyphosate contamination. Most planktonic microbiota responded to glyphosate exposure in terms of density, either positively or negatively. These results partially confirmed our hypotheses. In hypothesis i, a reduction over time in the richness and density of the groups was expected with the addition of the pesticide, but in some groups different responses were found. Regarding nanoflagellates, the results showed the positive effect of the presence of the pesticide over time, both for the autotrophic and heterotrophic fractions. This concurrent rise suggests that glyphosate altered bottom‐up drivers of microbial food webs, but the proximate mechanisms are likely different for the two fractions.

For autotrophic and heterotrophic flagellates, the addition of glyphosate had a positive effect. Autotrophic nanoflagellates, glyphosate seems to have acted as a source of phosphorus, stimulating their development [41], and also providing nutrients for other microorganisms [42]. Thus, for this group, the presence of glyphosate may have favored at least part of their populations. Regarding heterotrophic nanoflagellates, alterations in the heterotrophic fraction of nanoflagellates can have an indirect effect on the abundance of bacterioplankton, resulting in effects on the emission or sequestration of carbon in aquatic ecosystems. According to a study by Nathan et al. [43], pesticide can have a negative impact on bacterial abundance and carbonic anhydrase activity, affecting the process of carbon sequestration in the soil. In addition, glyphosate can have a direct effect on bacterial composition, and a single dose of glyphosate can change not only the abundance but also the composition of this community [44].

Ecosystem productivity can occur in two ways by the brown chain based on bacteria and the green chain based on the autotrophic fraction [45]. High primary production by large photoautotrophs produces dissolved organic matter that stimulates increased heterotrophic bacterial growth and, consequently, increases predator selectivity for fast‐growing heterotrophic bacteria at high productivity rates [46], this selective predation directly affects the carbon cycle, boosting the negative effect on bacteria due to the positive effect on flagellates.

Regarding total microfauna, the addition of the glyphosate resulted in a reduction in density, resulting in weak top‐down control, determined by the presence of glyphosate. Lethal effects of some herbicides have already been determined for freshwater metazoan groups, such as copepods and rotifers, affecting population dynamics and controlling survival, individual reproduction, and altering sex ratios [47]. The addition of glyphosate possibly impacted the water's chemical composition and, with this, led to changes in the planktonic microbiota (principally in T3). However, the recovery observed in T7 suggests either the negative effect is not persistent, which can be due to degradation of the compound over time or an adaptive response from the present species. Portinho et al. [17] also discuss that the zooplankton community can recover itself over time after a short exposure to glyphosate if the egg bank in the environment is viable.

In our study, the oscillatory pattern observed in density testate amoebae at the middle of the experiment (T3) suggested a non‐immediate effect of glyphosate, which may have resulted in the selection of more resistant species, leading to an increase in density at the end of the experiment (T7), favoring the dominance of more resistant individuals. Ndayishimiye et al. [48] showed that the pesticide‐related reduction in the richness and abundance of testate amoebae over time was associated with a decline in highly sensitive species. Although the pesticide did not eliminate species, i.e., affect richness, it may have affected physiological aspects such as reproduction rate, allowing those species less affected to develop rapidly over time. The increment in the density of testate amoebae may also be related to the increase in the concentration of organic carbon in the environment, mediating the processes of nutrient exchange between organisms through the assimilation of organic matter (e.g., dead organisms) [49], being important in making energy available to the environment [50]. Therefore, the testate amoebae community can be used to understand the resilience and recovery of the ecosystem after disturbance [51], since this community tends to recover over time after a disturbance such as the addition of pesticides.

Copepods also showed an oscillatory pattern; it also showed an oscillatory pattern. The effect of glyphosate on copepods can affect development and, at higher concentrations, the organisms may not survive to complete their life cycle [52]. According to Kulkarni et al. [53] life cycle characteristics and food selection, based on life stage and environmental conditions, can influence the amount of toxicant ingested and, consequently, differ in the effect of glyphosate on the community in question. Similarly, for rotifers, the increase in glyphosate inputs had important negative effects on active populations promoting a reduction in density [16].

Overall, the experiment showed that the different trophic groups responded in distinct ways to glyphosate addition, indicating that its effects are not uniform across biological communities. The results highlight a strong bottom‐up influence on community structure, with glyphosate functioning as a nutrient source. This enrichment increased the productivity and density of flagellates, potentially triggering imbalances associated with eutrophication and affecting intermediate trophic levels, such as testate amoebae. These protists play a key role as intermediaries between primary producers and top consumers, so fluctuations in their abundance can alter the energy flow available to higher trophic levels, sometimes reducing it, and at other times increasing it. These variations may also have been reflected in the dynamics of copepods, which exhibited patterns similar to those observed for testate amoebae, as well as in the overall reduction of total microfauna density. In summary, our results suggest that the presence of glyphosate can disrupt the coupling of energy flow along the food chain, compromising energy transfer to higher trophic levels.

The other groups of aquatic microbiota changed their abundance and richness only over time. This pattern may be related to the different successional processes followed by each experimental unit independently, regulated by the physical and chemical conditions and the availability of resources. Besides that, the interactions between the different species, within each microcosm, can also shape the communities over time [54].

### 4.2 Beta Diversity

The responses of the community composition to the different pesticide concentrations were observed for testate amoebae and cyanobacteria, with higher pesticide concentrations showing a more distinct composition. For testate amoebae, replacement (βrepl) was responsible for the changes in beta diversity. According to Ndayishimiye et al. [48], pesticides are correlated with the variability of testate amoeba communities, with ecological selection being imposed by abiotic and biotic variables, which can influence the organism's fitness and determine community composition. These findings reinforce the idea that more sensitive species are being replaced by more resistant ones that may be functionally redundant. Another explanation may lie in the composition results, which indicated that the pesticide was responsible for replacing testate amoebae species. The replacement of species over time can therefore reflect several non‐mutually exclusive processes, such as previously rare taxa that were below the detection limit at earlier sampling times (and became abundant enough to be detected by T7), excystment stages producing a sudden increase in detectable species, differential survival and growth rates led resistant taxa to increase, and stochastic differences among destructively sampled replicates making a species appear absent at one time and present at another.

For cyanobacteria, the differences of richness (βrich), responsible for the changes in beta diversity among treatments, explain why glyphosate can be used as a source of phosphorus, accelerating the growth of cyanobacteria and, consequently, improving their competitiveness [55]. Moreover, this occurrence of bloom conditions of some cyanobacteria species can result in an indirect impact on microflora considering that some of them are potentially toxic [56].

For the ciliates, there were changes in composition over time, due to the loss of species (βrich); i.e, there was a different composition between the times, suggesting responses to changes in resource availability, competition or predation along the course of the experiment. On the other hand, for rotifers, time was responsible for change through species replacement (βrepl). The influence of time on changes in beta diversity may be the result of changes in the original environmental conditions [57]. In this way, the community's responses to species replacement may be related to the presence of species that are more tolerant, where they persist for long periods in certain environments, especially those disturbed by anthropogenic actions [58]. In addition, the experimental time and its dynamics significantly influence the diversity of the planktonic community. Several studies have shown that environmental disturbances, the evolutionary history of different species [59], and the effects of experimental manipulation [60] also affect the communities diversity.

## 5 Conclusions

Our study provides important evidence on the impacts of glyphosate on planktonic microbiota, suggesting that glyphosate acts as both a resource and a stressor, depending on the concentration and on the biological groups. It was demonstrated distinct effects on microbiota communities and potential implications for the functioning of aquatic ecosystems. Primarily, it was evidenced that glyphosate could alter productivity, due to changes of basal levels. In addition, these possible changes in productivity may influence a larger scale, at ecosystem level, considering the biogeochemical cycle of carbon, and the fact that the food web can be not flowing well balanced and fluid as it should. As some levels are affected positively (e.g., autotrophic and heterotrophic nanoflagellates) and others oscillatory (e.g., testate amoebae), these distinct results may lead to disturbances in the balance of the ecosystem. Another important point to consider is the resilience of testate amoebae, which have managed to recover their density amid environmental changes, thus standing out as an important group for maintaining freshwater ecosystems. In summary, the results indicate a favoring of the autotrophic fraction and a possible limitation of heterotrophic communities, suggesting the predominance of control of the food web exercised from the base by primary producers. Although the effects of an environmental stressor are not always evident in studies involving multiple communities, this should reflect what occurs in natural environments, where several factors act simultaneously on organisms and communities. It is likely that, in many cases, interactions between communities and environmental conditions play a more relevant role in structuring them than the direct effect of glyphosate itself. Thus, such interactions can attenuate, mask, or even modify the direction of impacts, resulting in negative or positive responses depending on the community considered.

Therefore, future research should consider a broader timescale to enhance our understanding of glyphosate long‐term effects on communities, and to assess whether the strength of species interactions can outweigh the impact of glyphosate over time. Furthermore, studies examining the isolated effects of glyphosate on the most abundant microbial species in the environment, as well as on groups with similar functional traits, could provide more robust evidence and offer new insights into the observed patterns. Finally, it is important to protect aquatic environments from pesticides, as the responses of organisms are individual, and the impact on the distinct aquatic communities certainly determines changes in metabolism and ecosystem services.

## Author Contributions

**Melissa Progênio:** conceptualization, data curation, formal analysis, methodology, visualization, writing – original draft, writing – review and editing, validation, investigation. **Matheus Henrique Oliveira de Matos:** conceptualization, visualization, data curation, writing – original draft, writing – review and editing. **Edilaine Corrêa Leite:** methodology, writing – original draft, writing – review and editing. **Bianca Ramos de Meira:** conceptualization, writing – original draft, writing – review and editing. **João Vitor Bredariol:** data curation, writing – review and editing. **José Eduardo Gonçalves:** methodology, resources, visualization, writing – review and editing. **Pablo Augusto Poleto Antiqueira:** conceptualization, methodology, writing – review and editing. **Luiz Felipe Machado Velho:** conceptualization, methodology, visualization, supervision, writing – original draft, writing – review and editing.

## Funding

This work was supported by CAPES for granting their scholarship to author Melissa Progênio and CNPq for the research productivity grant to Luiz Felipe Machado Velho.

## Conflicts of Interest

The authors declare no conflicts of interest.

## Data Availability

The data that support the findings of this study are available on request from the corresponding author. The data are not publicly available due to privacy or ethical restrictions.
